# Commentary: Metabolic and hormonal changes after laparoscopic sleeve gastrectomy in pediatric population: an observational study

**DOI:** 10.3389/fsurg.2024.1177260

**Published:** 2024-03-27

**Authors:** Sveta M. Tokbergenova, Kaldybay S. Idrissov, Gulmira E. Bektenova, Ospan A. Mynbaev

**Affiliations:** ^1^ Department of Pediatrics, Khoja Akhmet Yassawi International Kazakh-Turkish University, Turkistan, Kazakhstan; ^2^ General Practitioner Department, South Kazakhstan Medical Academy, Shymkent, Kazakhstan; ^3^ N2 Pediatrics Department, South Kazakhstan Medical Academy, Shymkent, Kazakhstan; ^4^ Faculty of Biological & Medical Physics, Phystech BioMed School, Moscow Institute of Physics and Technology National Research University, Dolgoprudny, Russia; ^5^ New European Surgical Academy, Berlin, Germany

**Keywords:** laparoscopic sleeve gastrectomy, obesity, pediatric population, hormonal and metabolic changes, HbA1c analysis

A Commentary on Metabolic and hormonal changes after laparoscopic sleeve gastrectomy in pediatric population: an observational study By Alghamdi H, Asiri A, Alzahrani F, Alamri Z, AbdelQadir YH and Shah J (2022) Front. Surg. 9:1056458. doi: 10.3389/fsurg.2022.1056458

We read with great interest the paper by Alghamdi and colleagues (1) that studied nutritional biomarkers, glucose homeostasis indicators, blood lipids, hormones involved in the hypothalamic-pituitary-adrenal axis, and thyroid hormones before laparoscopic sleeve gastrectomy (LSGE) for managing severe obesity in children aged 5–14 years and repeated analysis 12 months after surgery. In comparison to baseline values, a significant reduction in circulatory C-peptide, hemoglobin, random blood glucose, and triglyceride concentrations was shown in postoperative observation (1). Subsequently, the authors concluded that 12 months following LSGE, there were improvements or the resolution of diabetes and hypertriglyceridemia among these children. However, blood cholesterol components, nutritional biomarkers, cortisol value, and thyroid profiles remained unchanged (1).

The growing population of obese or overweight people (2) is a worldwide concern of modern healthcare systems. Many projects were initiated by the WHO to focus governmental, business, and humanitarian organizations’ attention on this problem (3). Therefore, while reading such an interesting clinical observational study, we questioned whether the parameters of 5-year-old children and adolescents aged 14 could be comparable in these hormonal-metabolic parameters (HMPs). The authors described that 10 children were below or equal to 9 years old at the time of surgery and HbA1c analysis was stratified by gender, although such mixing of age-sensitive HMPs in a growing population decreases endpoints of such investigations. From the literature the higher rates of gluconeogenesis, on a body weight basis, and glucose production expressed per kilogram of body weight in children (8–9-year-old) than that of adolescents (14–16-year-old) were shown by Sunehag et al. (4). Vice versa, higher total plasma glucose Ra concentration was found in adolescents than in children (4). Analogously, age-specific reference curves for HbA1c levels in children and youth with type 1 diabetes (5) also differences in HMPs between children and adolescents can be found in the current literature.

There are differences between males and females during their intensively growing and pubertal periods for all studied HMPs. In these circumstances, if the authors include the laboratory references of normal-weight children and adolescents for males and females, there will be an explanation for HMPs’ changes related to surgery or the growing children. Another point for the question is that the authors (1) did not include preoperative and postoperative weight, height, and body mass index (BMI) parameters or dietary changes. However, they mentioned about in the discussion section. Subsequently, it is not clear the efficiency of surgical treatment of obesity in these children and adolescents.

It is well known that there are early and late complications of LSGE. However, the authors (1) did not describe the surgical technique in children and adolescents or its possible complications.

In this study (1), the authors aimed to evaluate HMPs’ changes in the pediatric population, but the aspects of this issue that we highlighted needed to be considered. The authors should not apply standards of this surgical treatment for adults to the pediatric population with a broad range of ages 5–14 years, including children and adolescents, as well as boys and girls in one group (1).

Nevertheless, to avoid this study’s shortcomings, the authors should consider the peculiar properties of the pediatric population depending on age groups in childhood and adolescent periods, as well as physiologic and hormonal differences in males and females during their active growing period.
